# Pore-tuning to boost the electrocatalytic activity of polymeric micelle-templated mesoporous Pd nanoparticles†
†Electronic supplementary information (ESI) available. See DOI: 10.1039/c8sc03911a


**DOI:** 10.1039/c8sc03911a

**Published:** 2019-03-12

**Authors:** Cuiling Li, Muhammad Iqbal, Bo Jiang, Zhongli Wang, Jeonghun Kim, Ashok Kumar Nanjundan, Andrew E. Whitten, Kathleen Wood, Yusuke Yamauchi

**Affiliations:** a School of Chemistry and Chemical Engineering , Beijing Institute of Technology , Beijing 100081 , China; b Key Laboratory of Eco-chemical Engineering , College of Chemistry and Molecular Engineering , Qingdao University of Science and Technology (QUST) , Qingdao 266042 , China; c International Center for Materials Nanoarchitectonics (WPI-MANA) , National Institute for Materials Science (NIMS) , 1-1 Namiki , Tsukuba , Ibaraki 305-0044 , Japan; d School of Chemical Engineering and Australian Institute for Bioengineering and Nanotechnology (AIBN) , University of Queensland , Brisbane , QLD 4072 , Australia . Email: y.yamauchi@uq.edu.au; e Australian Nuclear Science and Technology Organisation (ANSTO) , New Illawarra Rd, Lucas Heights , NSW 2234 , Australia; f Department of Plant & Environmental New Resources , Kyung Hee University , 1732 Deogyeong-daero, Giheung-gu , Yongin-si , Gyeonggi-do 446-701 , South Korea

## Abstract

Understanding how mesoporous noble metal architectures affect electrocatalytic performance is very important for the rational design and preparation of high-performance electrocatalysts. Herein, by using polymeric micelle-assembled structures as templates, mesoporous Pd nanoparticles with tunable porous constructions are synthesized by simply tuning the solvent compositions. The effect of porous Pd nanoparticles on the electrocatalytic performance is thoroughly studied. Their superior electrocatalytic activity can be attributed to the mass transport efficiency and open porous structures.

## Abstract 2

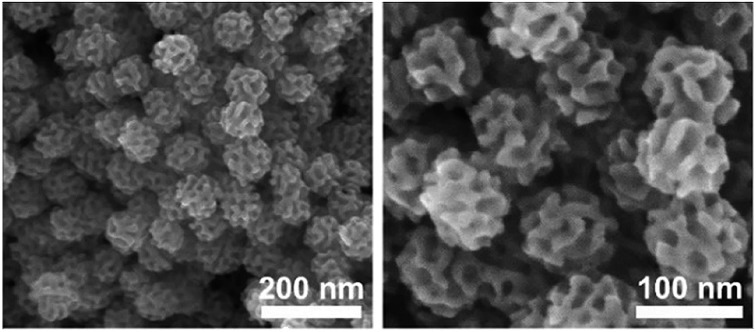
Understanding how mesoporous noble metal architectures affect electrocatalytic performance is very important for the rational design and preparation of high-performance electrocatalysts.

## Introduction

Porous features of mesoporous/nanoporous materials such as pore size and connectivity take a central role for a broad range of applications, including catalysis, sorption, and drug delivery.^1^–^5^ Therefore, rational control over the porous structures is fundamental and crucial to elucidate the relationship between the mesoscale structures and material performance. Decades of intense research efforts on the controlled synthesis of mesoporous materials show that the creation of new pore constructions is often dependent on the utilization of distinct surfactants.^6^–^11^ In most of cases, however, the compositions of the obtained materials are limited in metal oxides and carbon. Freely tuning the pore constructions in mesoporous metals is still challenging and has rarely been reported.

In general, the pore constructions can be guided by the surfactant packing parameter, *g* = *V*/*la*_0_, where *V* is the volume of the hydrophobic surfactant chains, *l* is the surfactant chain length, and *a*_0_ is the effective area of the hydrophilic head group of the surfactant molecule.^6^,^7^,^12^,^13^ A small *g* value favours the formation of mesophase with low surface curvature. A continuous decrease in the surface curvature results in the micelles transiting from spherical to rod-like and lamellar micelles, thus enabling the possibility to continuously tune pore structures. Polymeric micelle assemblies, which are formed by dissolving block copolymers in a mixed solvent with different solubilities to different blocks, have been developed to synthesize mesoporous metals.^14^–^18^ Considering the principle of the formation of polymeric micelles and the packing parameters, it is plausible to control the polymeric micelle structures by precisely tuning the solubilities of different blocks. The resulting porous metals with continuous pore constructions enable fundamental study of the effect of the porous structures on electrocatalytic performance.

Herein, by using polymeric micelle-assembled structures as templates, mesoporous Pd nanoparticles (abbreviated as meso-PdNPs) were prepared. By tuning the solvent compositions, the poly(styrene)-*b*-poly(ethylene oxide) (PS-*b*-PEO) polymeric micelles formed could be precisely controlled, resulting in mesoporous constructions varying from spherical mesopores to channel-like and lamellar mesopores. The samples were abbreviated as meso-PdNPs-*x*% in which *x* means the volume percentage (%) of tetrahydrofuran (THF) in the reaction solution.

## Experimental section

### Materials

Block copolymer (poly(styrene)-*b*-poly(ethylene oxide) (PS_(5000)_-*b*-PEO_(2200)_)) was purchased from Polymer Source, Inc. Ascorbic acid (AA), PdCl_2_ powder (99.0% purity) and hydrogen chloride (HCl, 35%) were obtained from Nacalai Tesque. The Nafion solution (∼5% in a mixture of lower aliphatic alcohols and water) and commercial Pd black used for electrochemical measurements were obtained from Sigma Aldrich. Before experiments, an 80 mM H_2_PdCl_4_ solution was prepared by dissolving 1 g of deep brown PdCl_2_ powder in 4.7 mL concentrated HCl solution (37%) and 65.8 mL deionized water. The solution was refrigerated for further use.

### Preparation of mesoporous Pd nanoparticles (meso-PdNPs) with continuously tunable porous structures

The synthesis of mesoporous Pd nanoparticles is based on an all-wet chemical reduction process. In a typical synthesis, 4 mg of PS-*b*-PEO was completely dissolved in THF. Then, deionized water, 80 μL of aqueous 2 M HCl solution, 0.25 mL of aqueous 80 mM H_2_PdCl_4_ solution, and 1 mL of aqueous 0.1 M AA solution were added to the above solution sequentially. A total volume of 2 mL was fixed for each synthesis, and the volume % of THF in the mixed solution was varied from 4% to 18% to control the solvent property. The mixed solution was further incubated under a water bath set at 50 °C for 10 h to yield a dark black solution. Finally, the samples were collected by centrifugation at 14 000 rpm for 15 min, followed by consecutive washing/centrifugation cycles with THF. This washing process could remove most of the block copolymer. Furthermore, the block copolymer was completely removed by thermally treating in air at 200 °C for 1 h. In correspondence with the experimental conditions, the samples obtained are abbreviated as meso-PdNPs-*x*%, in which *x* means the volume percentage (%) of THF in the reaction solution.

### Characterization

A field emission scanning electron microscope (SEM, HITACHI SU-8000) operating at an accelerating voltage of 5 kV was used to observe the morphology of Pd nanospheres. A high-resolution transmission electron microscope (HRTEM, JEOL JEM-2100F) operated at 200 kV was used to study the interior structure of the mesoporous nanoparticles. The hydrodynamic diameter (*D*_h_) of the polymeric micelles was measured with an Otsuka ELSA particle analyzer. Powder X-ray diffraction (XRD) measurements were conducted on a SmartLab X-ray diffractometer (Rigaku) at a scanning rate of 2 degrees per min with a Cu-Kα radiation (40 kV, 30 mA) source. Small-angle X-ray scattering (SAXS) measurements (Rigaku NANO-Viewer) were used to evaluate the pore-to-pore distance. The SAXS instrument used a Cu-Kα radiation (40 kV, 30 mA) source with a camera length at 700 mm.

### Electrochemical characterization

Electrochemical measurements were performed using a CHI 842B electrochemical analyzer (CHI Instrument, USA). The three-electrode cell consisted of a reference electrode (Hg/HgO electrode), a counter electrode (Pt wire) and a working electrode (glassy carbon electrode, GCE) modified by the catalysts. All catalysts were dispersed in ethanol to make the mass concentration of 1 mg mL^–1^. Then, 2 μL of the suspension was dropped onto the surface of the GCE. After the electrode was dried under air, 2 μL of Nafion solution (0.05 vol%) was then dropped onto the catalysts. The electrode was left to become thoroughly dried up under room temperature for further electrochemical measurements. Ethanol electro-oxidation measurements were carried out in 1.0 M KOH containing 1.0 M ethanol.

## Results and discussion

**Fig. 1:**
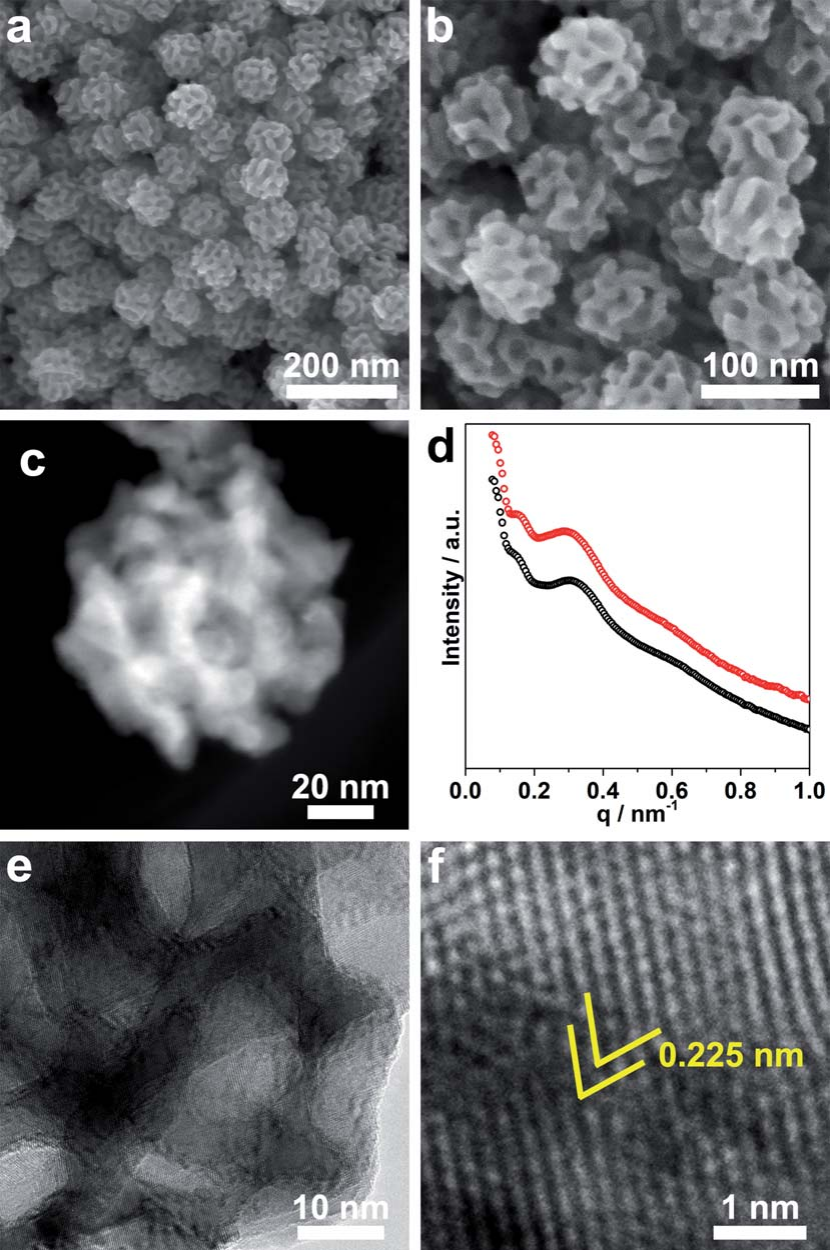
(a, b) SEM images of the obtained meso-PdNPs-4%. (c) HAADF-STEM image of one typical meso-PdNP-4%. (d) SAXS patterns of the meso-PdNPs-4% before (black dots) and after (red dots) removal of the block copolymers. (e, f) HRTEM images of the meso-PdNPs-4%.

As a typical product, the structure of meso-PdNPs-4% was characterized by SEM and TEM. The obtained porous nanoparticles were uniform, with a size of 75 ± 5 nm (Fig. 1a, b and S1a, b†). The thickness of their pore walls and the average pore sizes were estimated to be 7.3 ± 0.3 nm (Fig. S1c†) and 12.6 ± 0.8 nm (Fig. S1d†), respectively, by statistically analyzing more than 300 nanoparticles. The high-annular dark-field scanning TEM (HAADF-TEM) image clearly showed the porous structure of one typical nanoparticle (Fig. 1c). The periodicity in the porous structures was confirmed by using SAXS. The most-intense peak centered at 0.31 nm^–1^, corresponding to the pore-to-pore distance of 20.4 nm, which was consistent with the aforementioned results (*i.e.*, 7.3 + 12.6 = 19.9 nm), was distinctly observed from the SAXS profile (Fig. 1d), showing the ordering of mesostructures. The remaining peaks in the low *q* range were derived from the uniform size of porous PdNPs. The SAXS profiles of the samples before and after removing the block copolymers showed similar diffraction patterns (Fig. 1d), indicating the porous structure was stable without any structural collapse during the removal of pore-directing agents.

The crystalline phase of the obtained sample was confirmed by wide-angle XRD. The distinct five diffraction peaks centered at around 40.0°, 46.4°, 67.9°, 82.0° and 86.7° corresponded to the (111), (200), (220), (311) and (222) planes of face-centered cubic (fcc) Pd (Fig. S2†). The average grain size calculated using the Scherrer equation was 16.9 nm, which was larger than the thickness of the porous frameworks (7.3 ± 0.3 nm). HRTEM was then employed to further investigate the crystallinity of the obtained mesoporous Pd nanoparticles (Fig. 1e and f). In a highly magnified domain, the lattice fringes with a constant *d* spacing of 0.225 nm could be assigned to the (111) diffraction planes of fcc Pd crystals (Fig. 1f). The selected area electron diffraction (SAED) patterns of different nanoparticles showed the presence of both single and polycrystals (Fig. S3†), supporting the existence of a larger grain size than that of the pore wall thickness. Although diverse methods have been developed to synthesize mesoporous materials, it has been extremely difficult to create highly crystallized pore walls, in part because crystallinity brings about distortion of pore walls, thus resulting in the collapse of the porous structures.^19^,^20^ Herein, the orientated integration of Pd atoms probably took place at the surfaces of the Pd nanocrystals, resulting in single-crystal Pd nanoparticles with a porous structure. Detailed discussion of the time-dependent TEM images is given later in the text.^21^ The present work shows the possibility of creating highly crystalized pore walls by surpassing the crystal growth over a facile soft template, which is valuable for creating many mesoporous crystallized metals/alloys with wide applications.

**Fig. 2:**
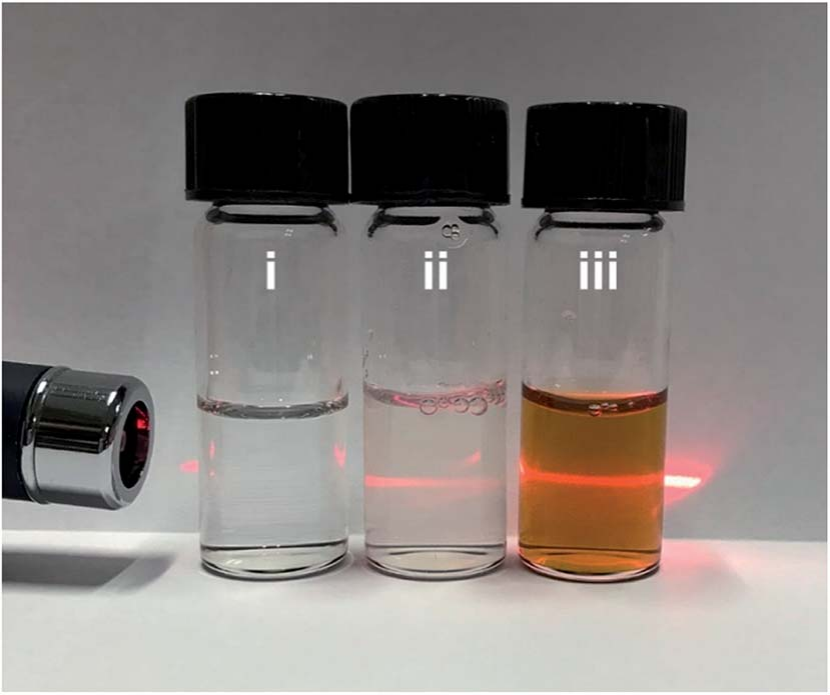
Photo showing the Tyndall effect in solutions with different compositions: (i) PS-*b*-PEO dissolved in THF, (ii) PS-*b*-PEO dissolved in H_2_O mixed with 4% THF, and (iii) PS-*b*-PEO dissolved in H_2_O mixed with 4% THF + H_2_PdCl_4_.

Using our method, meso-PdNPs with tunable pore constructions have been achieved, which relies on the polymeric micelle-assembled structure changing with the solvent compositions. The formed micelles can serve as pore-directing agents to guide the growth of Pd nanostructures. In our experiments, the block copolymer, PS-*b*-PEO, was first dissolved in THF, which is clear and transparent (see solution (i) in Fig. 2). After the addition of deionized H_2_O or the aqueous solution of H_2_PdCl_4_, the polymeric micelles were triggered to form under variation of the solvent polarity, resulting in a transparent solution (see the Tyndall effect in solutions (ii) and (iii) in Fig. 2). The change of the micelle constructions before and after mixing with the metal precursor (herein, H_2_PdCl_4_) in the solution was then directly characterized by small-angle neutron scattering (SANS).

**Fig. 3:**
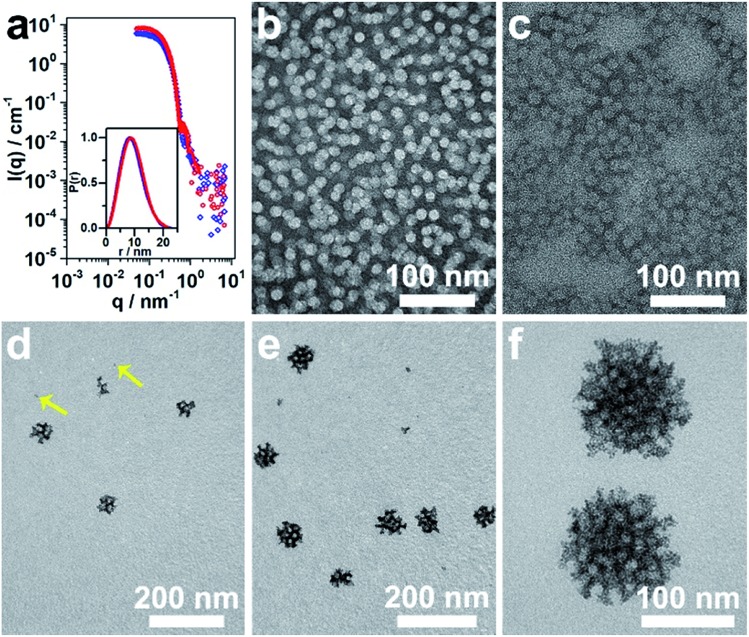
(a) SANS data of micelles in 4% THF/D_2_O (red dots) and 9% THF/D_2_O (blue diamonds), which were fitted to the polydisperse core–shell model plotted as lines. Inset: the probability distribution functions extracted from the data, which are consistent with spherical particles in solution. (b, c) Typical TEM images of the micelles stimulated without (b) and with (c) the addition of H_2_PdCl_4_. (d–f) TEM images showing the growth process of meso-PdNPs-4%.

The micelles were prepared following the typical experimental procedure except for using a deuterated solvent (herein, D_2_O) to enhance the scattering contrast. The SANS data were collected on the Quokka instrument^22^ at the Australian Nuclear Science and Technology Organisation and reduced and modeled using macros in Igor Pro.^23^ The obtained data from the polymeric micelles at 4% and 9% (vol%) of THF in D_2_O, referred to as 4% THF/D_2_O and 9% THF/D_2_O, respectively, before and after incorporating with metal precursor are presented in Fig. 3a and S4a.† As the extracted probability distribution functions for both the polymeric micelle samples were consistent with spherical particles, spherical core–shell particle fits were performed on the data (Fig. 3a), and the resultant parameters are shown in Table S1.† The spherical micelles extracted from the full scattering curves confirmed a core radius of 4.4 nm and shell thickness of 4.0 nm for the micelles stimulated only by D_2_O. Based on the present conditions and previous studies, the micelles with PS block as core and PEO block as shell were well formed here. The core–shell construction could be further supported by the core radius expansion (to be *ca.* 5.0 nm) and shell thickness decrease (*ca.* 2.9 nm) when the THF amount was increased from 4% to 9% (Table S1†); probably ascribed to the swollen effect of the THF to the PS blocks.^14^ Once the H_2_PdCl_4_ was added, both TEM and SANS data indicated that the micelles self-assembled into more elongated structures (Fig. 3b, c, S4 and Table S1†).

The confined growth manner of the Pd nanocrystals under polymeric micelles is particularly important to create porous structures. The water-coordinated Pd ions are thought to interact with the shells of the micelles through hydrogen bonding. After addition of reducing agent, the Pd metal constructs the pore walls, which envelop the micelles gradually. Therefore, the growth manner of Pd nanocrystals was investigated by TEM. The tiny Pd crystals (as indicated by arrows in Fig. 3d) were first formed and accumulated. Further growth of the Pd nanocrystals resulted in curved surfaces even when the nanocrystals were less than 20 nm in size (Fig. 3d and e). With aggregation of enough micelles, the crystals quickly reached the maximum size (Fig. 3f).

**Fig. 4:**
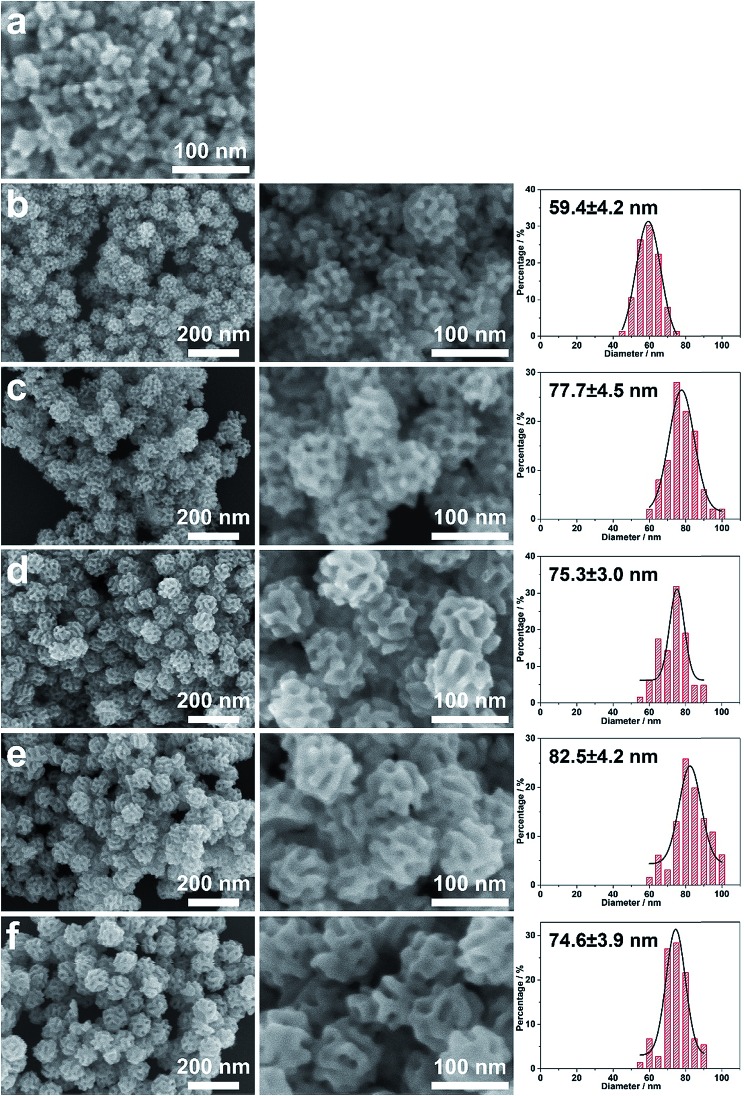
Typical SEM images of the as-prepared meso-PdNPs under the typical conditions with various Pd concentrations: (a) 1.6 mM, (b) 3.2 mM, (c) 4.8 mM, (d) 9.6 mM, (e) 12.8 mM, and (f) 16.0 mM, respectively. The histograms on the right side show the distribution of the corresponding particle sizes.

The concentration of the metal precursor may greatly influence the accumulating manner of the crystals, thereby determining the pore constructions.^24^,^25^ Therefore, the dependence of porous structure on the Pd concentration was studied under the typical synthetic conditions (Fig. 4). No porous products could be formed when the Pd concentration was quite low (1.6 mM) (Fig. 4a), which could be ascribed to the insufficient amount of the Pd precursor. On the other hand, porous structures appeared when the Pd concentration was increased to 3.2 mM (Fig. 4b) and became well defined at a Pd concentration of 4.8 mM (Fig. 4c). With higher concentrations of Pd (*i.e.*, 9.6 mM, 12.8 mM, and 16.0 mM), spherical porous structures were formed throughout but with a slight difference in particle sizes (Fig. 4d–f).

**Fig. 5:**
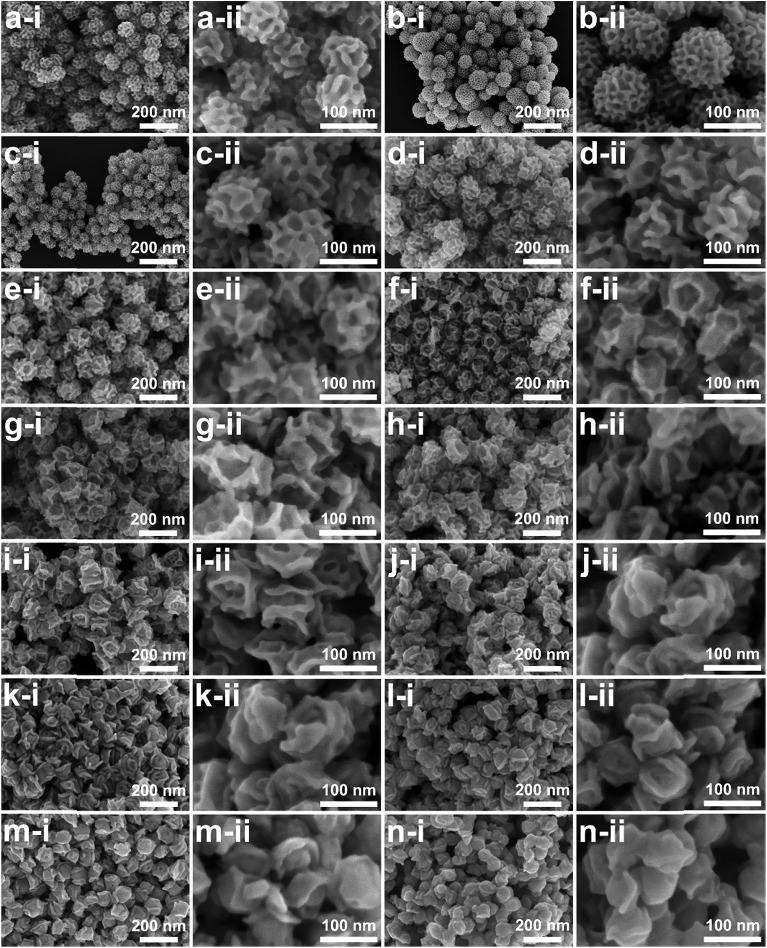
Typical SEM images at different magnifications (i and ii) of the obtained Pd nanoparticles under typical conditions by changing the THF volume concentrations: (a) 4%, (b) 5%, (c) 6%, (d) 7%, (e) 8%, (f) 9%, (g) 10%, (h) 11%, (i) 12%, (j) 13%, (k) 14%, (i) 15%, (l) 16%, (m) 17%, and (n) 18%, respectively.

The deformation of micelles is also essential for control over the pore constructions based on the geometric factors of the surfactant packing parameters.^6^,^7^,^12^,^26^ As has been thoroughly revealed, the surfactant concentration and the solvent composition are the two key factors influencing the micelle structures. Herein, the influence of the block copolymer concentration was first studied. THF is a good solvent of the hydrophobic PS block as it can enter the hydrophobic core of the micelle and increase its volume. As the volume of the hydrophobic core increases, micelles with a lower curvature surface are preferred, usually resulting in micelle deformation from spherical to rod-like and lamellar structures. Motivated by the dominating influence of the amount of THF on the micelle structures, the dependence effect of the porous structures on THF concentration was investigated. For this, the resultant porous structures obtained by gradually increasing the THF concentration from the optimal (4%) to a maximum value (18%) were studied by SEM (Fig. 5). The typical porous structure first expanded to be a more open structure when the THF amount increased from 4% to 9% (Fig. 5a–f). This can be explained by the expansion of micelle cores with increasing THF amount, which is consistent with the TEM and SANS measurements. Further increase of the THF amount (10% to 17%) resulted in the micelles changing from spherical to rod-like micelles, thus bringing out channel-like pores (Fig. 5g–m and S5†). At a THF concentration of 18%, mesoporous Pd nanoparticles with a layered structure were obtained (Fig. 5n and S6).

Pd has aroused intensive research interest for commercializing direct ethanol fuel cells (DEFCs) due to its relative abundance, low cost, and outstanding catalytic activity and durability toward the ethanol oxidation reaction (EOR).^27^–^34^ Taking advantage of the continuously tunable pore constructions, it is plausible to study the relationship between electrocatalytic performance and pore construction. Therefore, the as-prepared mesoporous Pd with different pore constructions (spherical pores (meso-PdNPs-4%) and channel-like pores (meso-PdNPs-12%)) were selected and evaluated for electrocatalyzing the EOR. Commercially available Pd black (PdB) was used as the reference for comparison.

**Fig. 6:**
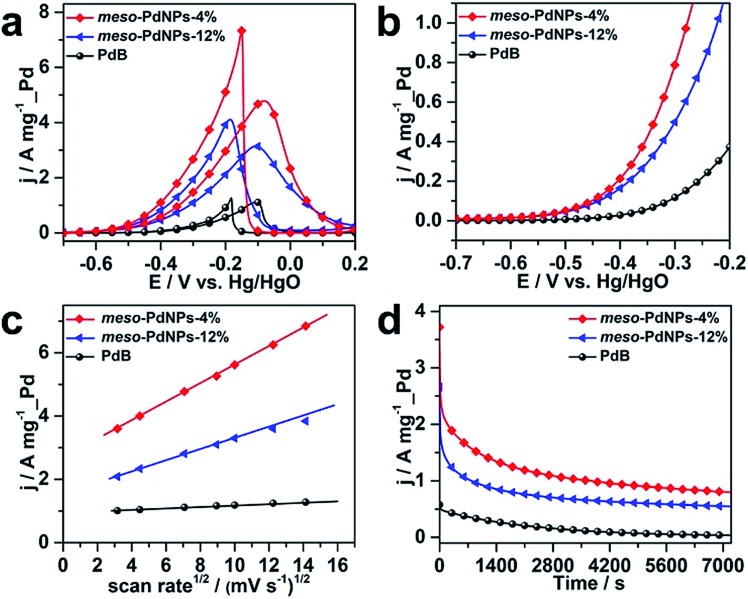
(a) Mass-normalized cyclic voltammograms (CVs), (b) linear sweep voltammograms (LSVs), and (d) chronoamperometric curves (recorded at –0.2 V) for EOR in 1.0 M NaOH containing 1.0 M C_2_H_5_OH. (c) The relationship of peak current density *versus* the square root of the scan rate. The CV and LSV curves were obtained at a scan rate of 50 mV s^–1^ and 1 mV s^–1^, respectively.

The typical cyclic voltammogram (CV) curves of meso-PdNPs-4%, meso-PdNPs-12%, and PdB obtained in 1.0 M NaOH containing 1.0 M C_2_H_5_OH electrolyte at a scan rate of 50 mV s^–1^ are displayed in Fig. 6a. To delineate the catalytic performance, the current densities were normalized by the Pd loading amount to get the mass activity. The mass activity of meso-PdNPs-4% is 4.78 A mg^–1^_Pd, which is 1.52 and 4.19 times higher than that of meso-PdNPs-12% (3.15 A mg^–1^_Pd) and PdB (1.14 A mg^–1^_Pd), respectively (Fig. 6a). The anodic current of the meso-PdNP catalysts rapidly increased with a positive scan. In sharp contrast, commercial PdB showed moderate activity toward EOR in the whole potential range (Fig. 6b and Table 1). Furthermore, the catalytic performance of the as-prepared meso-PdNPs toward EOR outperformed most of the reported Pd-based electrocatalysts, as summarized in Table S2.† The enhanced catalytic activity of mesoporous Pd nanoparticles prompts us to explore the reaction mechanism. The forward CVs of the EOR at different scan rates are shown in Fig. S7,† and the corresponding relationship between the peak current density and the square root of scan rate is shown in Fig. 6c. The oxidation current density is proportional to the square root of the scan rate, suggesting that the oxidation of ethanol catalyzed by all the catalysts is controlled by a diffusion process. The slope is an indicator of the diffusion efficiency. The larger slope value obtained at the meso-PdNPs-4% electrocatalyst implied fast electron and mass transfer for the Pd catalysts (Fig. 6c).^35^–^38^ The comparison of the current density of ethanol oxidation at –0.2 V was carried out at room temperature (Fig. 6d). The oxidation current density of the meso-PdNPs-4% kept exceeding that of meso-PdNPs-12% and PdB for the entire timescale. After the long-term stability measurement, the meso-PdNPs could retain their catalytic activity toward EOR (Fig. S8†), supporting the good stability of the mesoporous constructions. Benefiting from the tunability of pore constructions, the correlation between electrocatalytic performance and pore constructions is clearly revealed.

**Table 1:** Onset potentials and current densities at –0.4 V and –0.3 V of the meso-PdNPs-4%, meso-PdNPs-12%, and PdB catalysts during the LSV tests

Catalyst	*E*_onset_ (V *vs.* Hg/HgO)	*j* at –0.4 V (A mg^–1^_Pd)	*j* at –0.3 V (A mg^–1^_Pd)
meso-PdNPs-4%	–0.55	0.22	0.79
meso-PdNPs-12%	–0.55	0.16	0.50
PdB	–0.48	0.026	0.117

## Conclusions

Mesoporous Pd nanoparticles have been synthesized by a polymeric micelle assembly approach. The approach enables tuning of pore constructions by changing the solvent compositions. Benefiting from the flexibility of pore constructions, the effect of porous constructions on electrocatalytic performance was studied by using the ethanol oxidation reaction as the model reaction. The present work paves ways for the controlled synthesis of other porous materials with tunable pore constructions and towards promising practical applications.

## Conflicts of interest

There are no conflicts to declare.

## Supplementary Material

Supplementary information
