# Supplementary material for: Pore-tuning to boost the electrocatalytic activity of polymeric micelle-templated mesoporous Pd nanoparticles
Source: Chem Sci. 2019 Mar 12;10(14):4054–61. doi: 10.1039/c8sc03911a (PMC6457336; doi:10.1039/c8sc03911a)
Supplement: Supplementary file 1 [file SC-010-C8SC03911A-s001.pdf]

This journal is © The Royal Society of Chemistry 2019

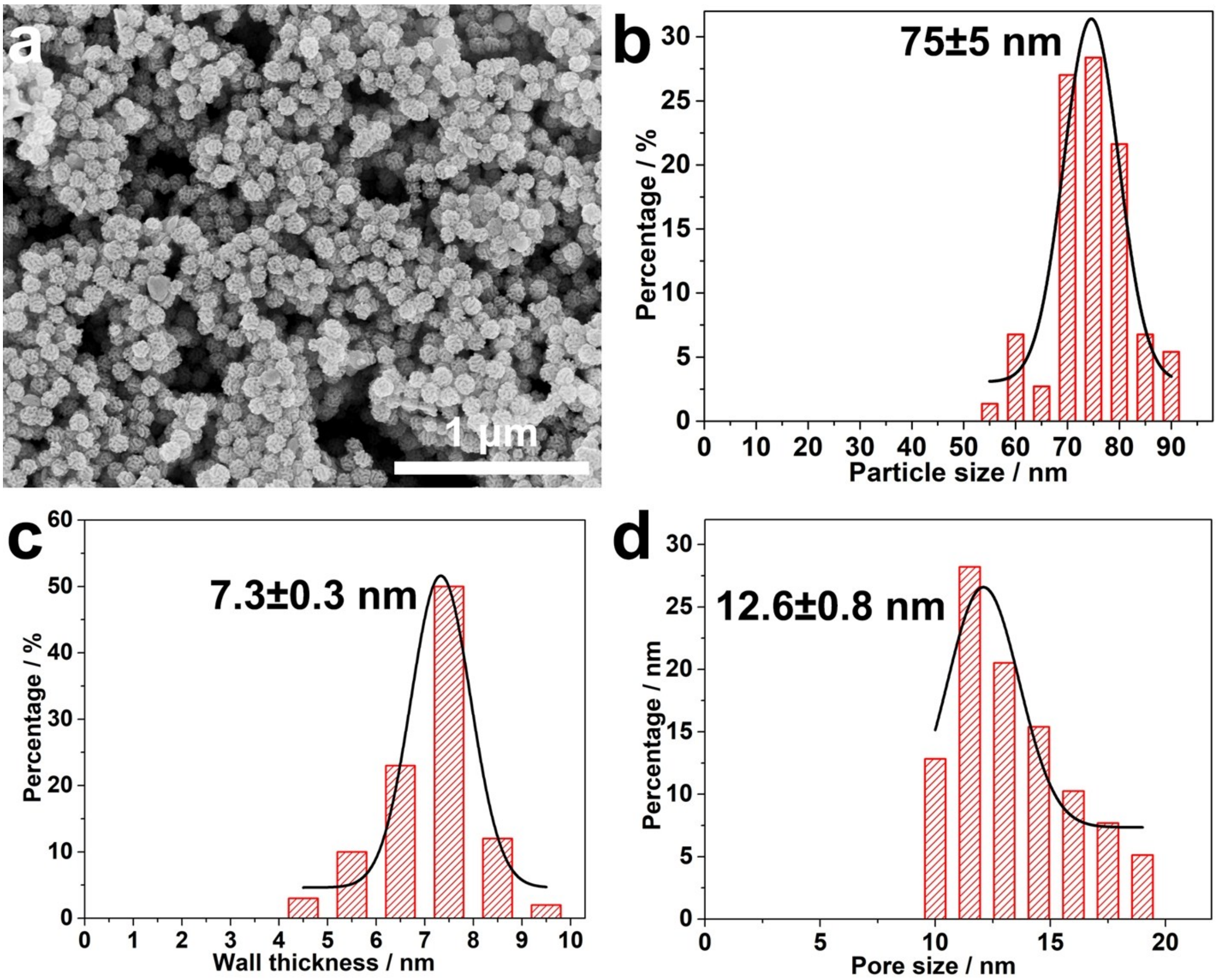

Figure S1. (a) Low-magnification SEM image of meso-PdNPs-4%. The histograms of (b) particle size, (c) wall thickness, and (d) pore size distributions of meso-PdNPs-4%.

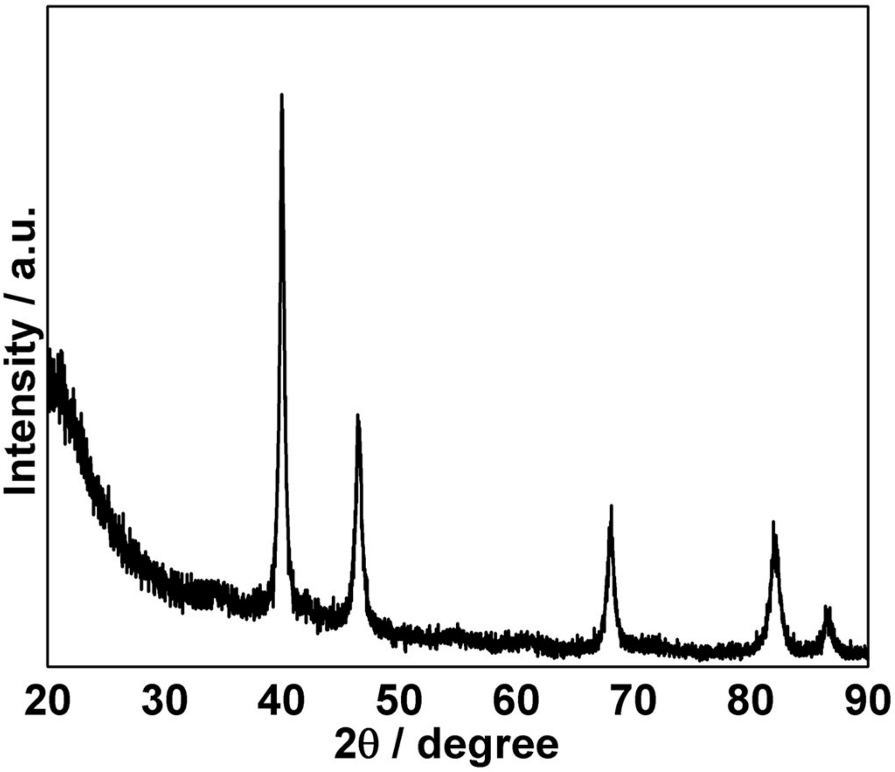

Figure S2. XRD pattern of the meso-PdNPs-4%.

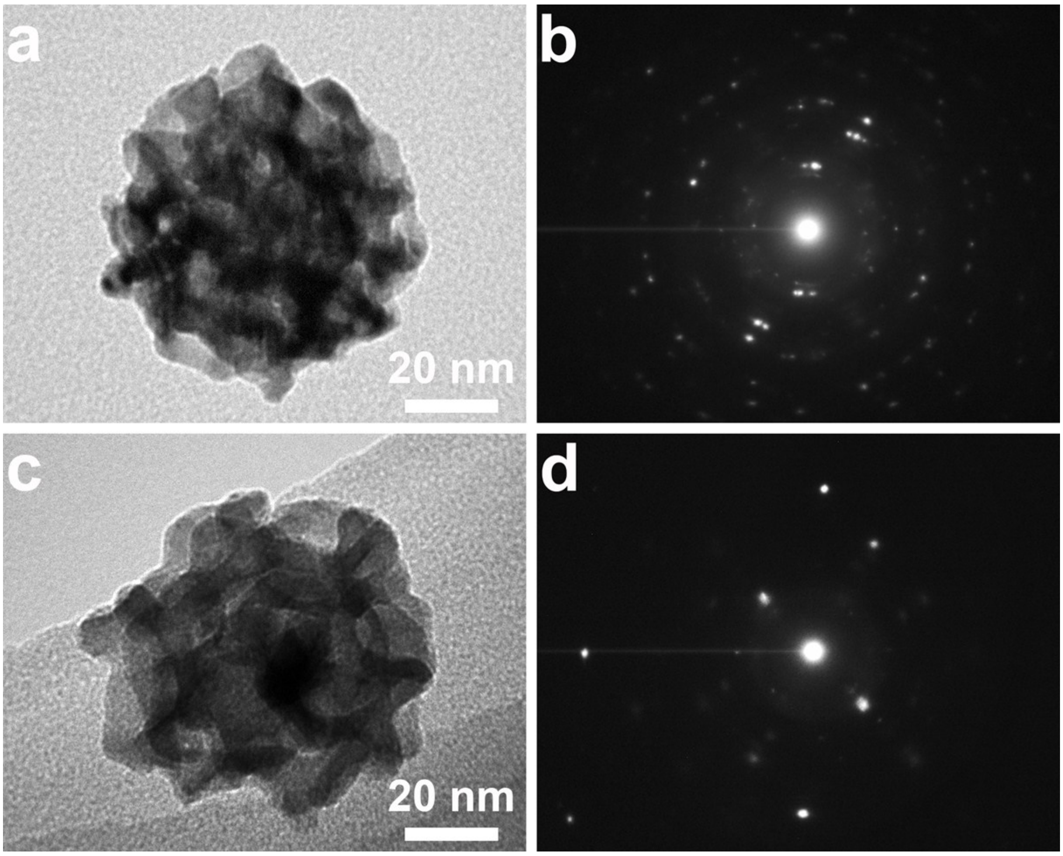

Figure S3. (a, c) Typical TEM images and (b, d) the corresponding SAED patterns of one individual meso-PdNP-4% illuminating the high crystallinity.

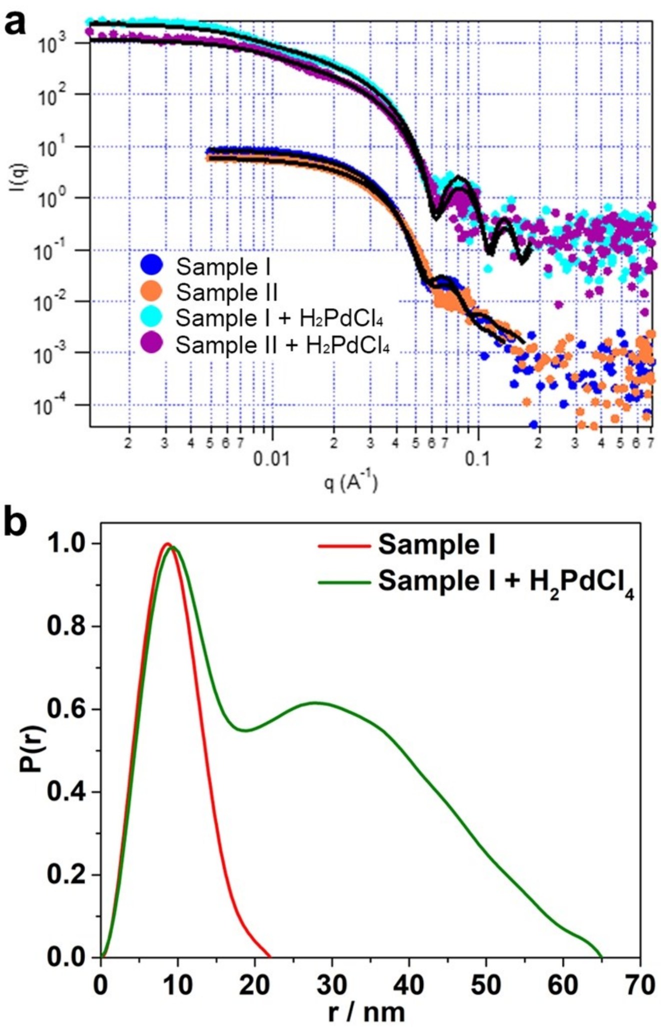

Figure S4. (a) Small angle neutron scattering data for polymeric micelles by dissolving PS-*b*-PEO in 4%THF/D_2O (Sample I), 9%THF/D_2O (Sample II), Sample I + H_2PdCl_4, and Sample II + H_2PdCl_4, respectively. Fits to the data represent a polydisperse core-shell model for Samples I and II without H_2PdCl_4 and a cylinder model of uniform scattering length density for Samples I and II with H_2PdCl_4. Further details about the models used to fit the data are given below **Table S1**. (b) The probability distribution functions extracted from the data, which are consistent with spherical shaped particles in solution for Sample I. Sample I + H_2PdCl_4 shows that the polymer self-assembles into much larger structures in solution on addition of the metal precursor.

Table S1. Parameters extracted from fits to geometric shapes using NIST analysis macros in Igor Pro.

Samples	Radius[c] [Å]	Core radius[d] [Å]	Shell thickness[d] [Å]	Cylinder radius[e] [Å]	Cylinder length[e] [Å]
Sample I[a]	82.5 ± 0.1	44.1 ± 0.1	40.2 ± 0.1		
Sample II[b]	78.6 ± 0.1	50.4 ± 0.1	29.3 ± 0.1		
Sample I + H_2PdCl_4				63.0 ± 0.1	690 ± 12
Sample II + H_2PdCl_4				61.7 ± 0.1	547 ± 7

[a]Sample I was prepared by dissolving 4 mg of PS-*b*-PEO in 0.08 mL THF and 1.92 mL D_2O, *i.e.* 4%THF/D_2O.

[b]Sample II was prepared by dissolving 4 mg of PS-*b*-PEO in 0.18 mL THF and 1.82 mL D_2O, *i.e.* 9%THF/D_2O.

[c]Data fit with a polydisperse sphere model of uniform scattering length density.

[d]Data fit with a polydisperse core-shell particle model with different scattering length densities for core and shell.

[e]Data fit with a cylinder model.

Notes for Figure S4 and Table S1:

The simplest model that can adequately describe the scattering from Samples I and II is a polydisperse sphere model, which assumes a uniform scattering length density throughout the micelles. The single parameter extracted from such a model is the radius of the spheres, which is found to decrease from 82.5 Å to 78.6 Å on increasing the concentration of THF. Since PS-*b*-PEO assembled into a core-shell structure, a polydisperse core-shell model was then applied to model the difference between the PS core and the outer PEO shell and reasonable values for the core and shell were extracted from fits, although only a small improvement was seen in overall fit quality. The core radius was found to increase as expected when the amount of THF in the sample was increased. Once the metal precursor was added in solution, the SANS data showed that significantly larger micellar structures were present. The data were fitted with a cylinder model of uniform density as the simplest model that could describe the data. Other models such as a mix of cylinders and spheres were also found to give reasonable fits to the data (not shown).

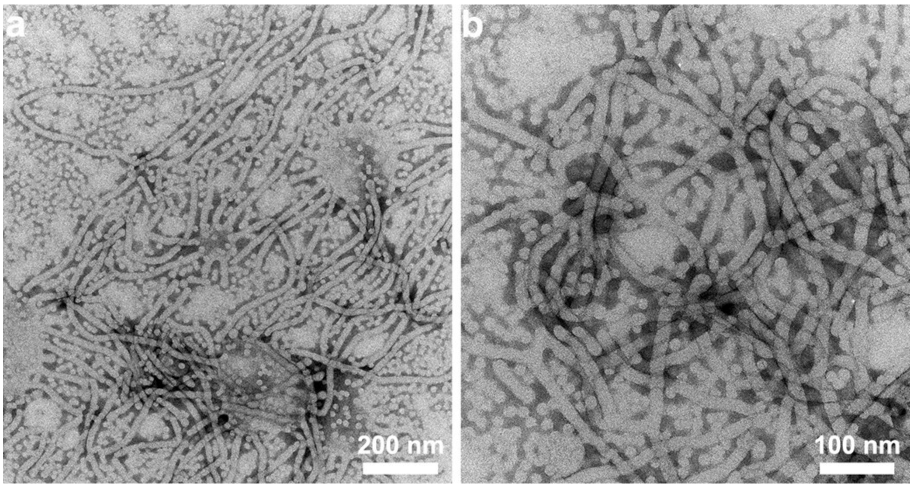

Figure S5 a) Low- and b) high-magnification TEM images of the polymeric micelles formed by dissolving PS-*b*-PEO in H_2O mixed with 12%THF without addition of H_2PdCl_4.

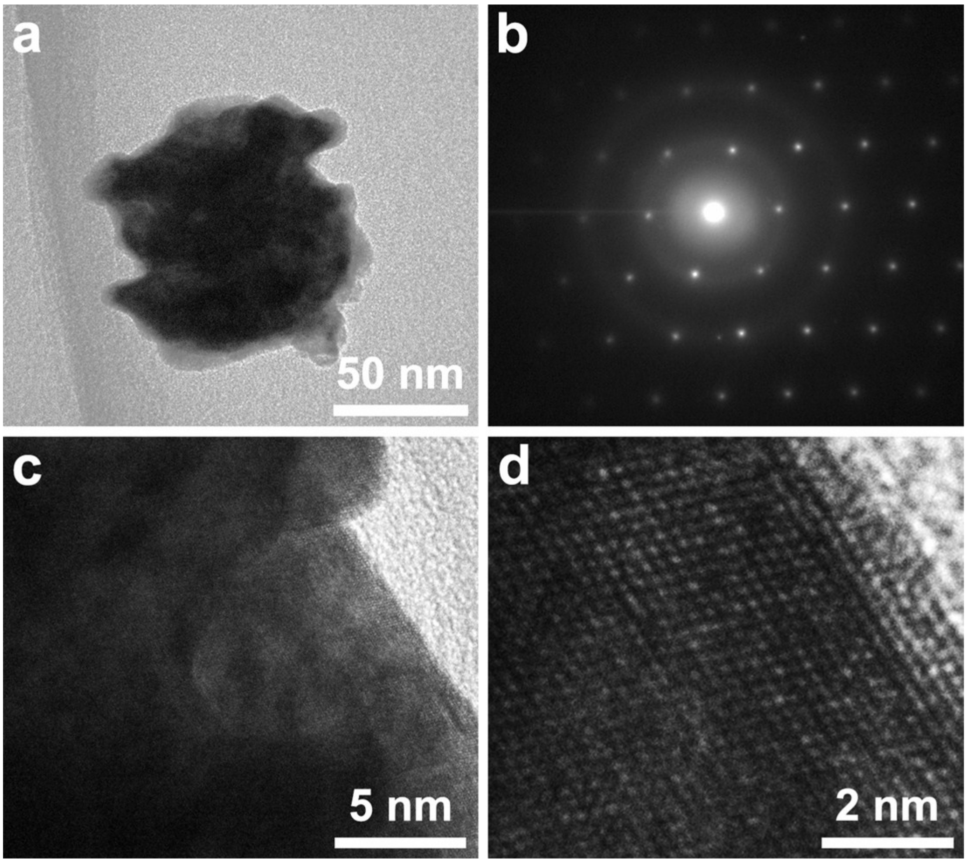

Figure S6 a) TEM, and c,d) HRTEM images of the as-prepared meso-PdNPs-18%. Panel b) shows the SAED pattern of the single nanoparticle shown in panel a).

Table S2. Comparison of the EOR mass activity catalyzed by different Pd-based electrocatalysts.

Catalysts	Electrolyte	Scan rate ($mV\ s^{-1}$)	Mass activity ($A\ mg^{-1}\_Pd$)	References
Mesoporous Pd nanoparticles	1M NaOH + 1M C_2H_5OH	50	4.78	Present work
PdCo nanotube arrays	1M NaOH + 1M C_2H_5OH	50	1.50	S1
Ultrathin Pd nanomesh	1M NaOH + 1M C_2H_5OH	50	5.40	S2
PdAg bimetallic alloy networks	1M NaOH + 1M C_2H_5OH	50	1.97	S3
Ultrafine FePd nanoalloys-MWCNTs	1M NaOH + 1M C_2H_5OH	50	1.20	S4
Pd-Pt bimetallic heterostructures	1M NaOH + 1M C_2H_5OH	50	12.65	S5
Pd-CNTs	1M NaOH + 1M C_2H_5OH	50	2.86	S6
Pd-Ni-P	1M NaOH + 1M C_2H_5OH	50	4.95	S7
Flower-like PdAuCu-rGO-CNT framework	1M NaOH + 1M C_2H_5OH	50	2.35	S8
PdPt bimetallic alloy nanowires	0.1M NaOH + 1M C_2H_5OH	50	~0.9	S9
Pd-Ni-P electrocatalysts	0.1M NaOH + 1M C_2H_5OH	10	~0.11	S10

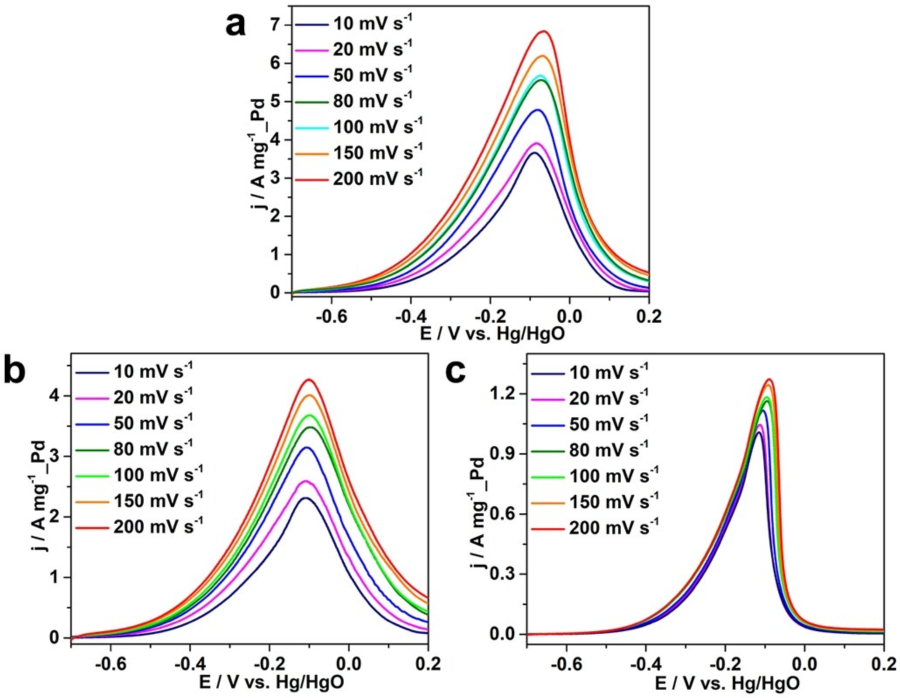

Figure S7. CVs of the EOR catalyzed by a) meso-PdNPs-4%, b) meso-PdNPs-12%, and c) PdB in 1.0 M NaOH containing 1.0 M C_2H_5OH at different scan rates.

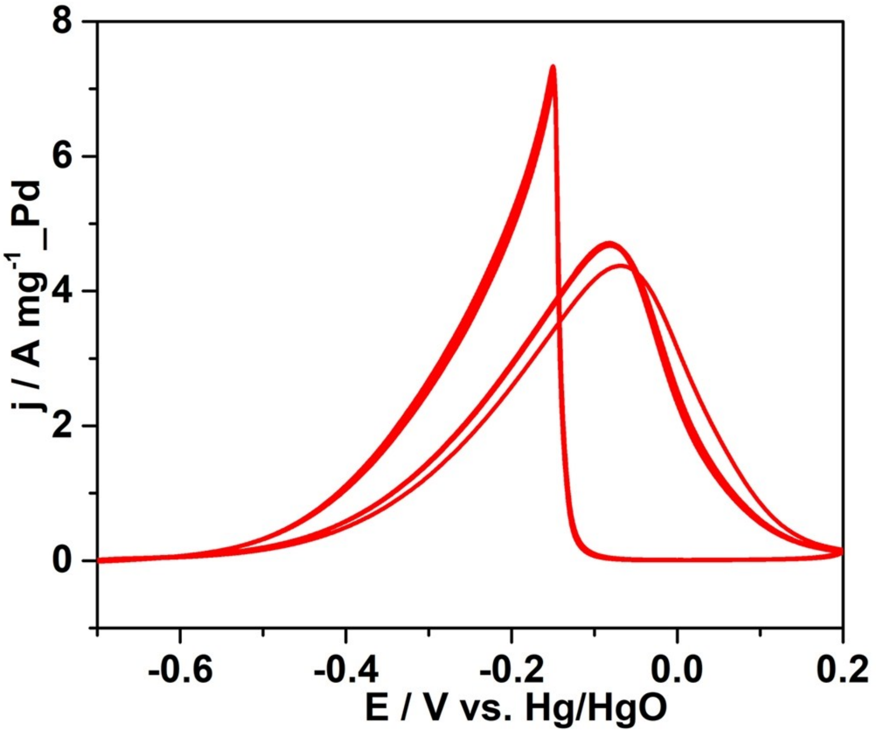

Figure S8. Typical CV curves (10 successive cycles) of EOR catalyzed by meso-PdNPs-4% in 1.0 M NaOH containing 1.0 M C_2H_5OH after durability test for 2 h.

References

[S1] A.-L. Wang, X.-J. He, X.-F. Lu, H. Xu, Y.-X. Tong, G.-R. Li, *Angew. Chem. Int. Ed.* **2015**, 54, 3669-3673.

[S2] J. Ge, P. Wei, G. Wu, Y. Liu, T. Yuan, Z. Li, Y. Qu, Y. Wu, H. Li, Z. Zhuang, X. Hong, Y. Li, *Angew. Chem. Int. Ed.* **2018**, 57, 3435-3438.

[S3] S. Fu, C. Zhu, D. Du, Y. Lin, *ACS Appl. Mater. Interfaces* **2015**, 7, 13842-13848.

[S4] Y. Wang, Q. He, J. Guo, J. Wang, Z. Luo, T. D. Shen, K. Ding, A. Khasanov, S. Wei, Z. Guo, *ACS Appl. Mater. Interfaces* **2015**, 7, 23920-23931.

[S5] J. Fan, K. Qi, L. Zhang, H. Zhang, S. Yu, X. Cui, *ACS Appl. Mater. Interfaces* **2017**, 9, 18008-18014.

[S6] J. Zhang, Y. Cheng, S. Lu, L. Jia, P. K. Shen, S. P. Jiang, *Chem. Commun.* **2014**, 50, 13732-13734.

[S7] L. Chen, L. Lu, H. Zhu, Y. Chen, Y. Huang, Y. Li, L. Wang, *Nat. Commun.* **2017**, 8, 14136.

[S8] M. Wang, Z. Ma, R. Li, B. Tang, X.-Q. Bao, Z. Zhang, X. Wang, *Electrochim. Acta* **2017**, 227, 330-344.

[S9] C. Zhu, S. Guo, S. Dong, *Adv. Mater.* **2012**, 24, 2326-2331.

[S10] R. Jiang, D. T. Tran, J. P. McClure, D. Chu, *ACS Catal.* **2014**, 4, 2577-2586.
